# Identification and Validation of a Tumor Microenvironment-Related Gene Signature in Hepatocellular Carcinoma Prognosis

**DOI:** 10.3389/fgene.2021.717319

**Published:** 2021-11-26

**Authors:** Changjing Huang, Chenyue Zhang, Jie Sheng, Dan Wang, Yingke Zhao, Ling Qian, Lin Xie, Zhiqiang Meng

**Affiliations:** ^1^ Minimally Invasive Therapy Center, Fudan University Shanghai Cancer Center, Shanghai, China; ^2^ Department of Oncology, Shanghai Medical College, Fudan University, Shanghai, China; ^3^ Department of Integrated Therapy, Fudan University Shanghai Cancer Center, Shanghai, China; ^4^ Cancer Institute, Fudan University Shanghai Cancer Center, Fudan University, Shanghai, China

**Keywords:** hepatocellular carcinoma, tumor microenvironment, prognosis, EPO, BIRC5, SPP1

## Abstract

**Background:** Hepatocellular carcinoma (HCC) is a typical inflammatory-related malignant tumor with complex immune tolerance microenvironment and poor prognosis. In this study, we aimed to construct a novel immune-related gene signature for the prognosis of HCC patients, exploring tumor microenvironment (TME) cell infiltration characterization and potential mechanisms.

**Methods:** A total of 364 HCC samples with follow-up information in the TCGA-LIHC dataset were analyzed for the training of the prognostic signature. The Least Absolute Shrinkage and Selector Operation (LASSO) regression based on the IRGs was conducted to identify the prognostic genes and establish an immune risk signature. The immune cell infiltration in TME was estimated *via* the CIBERSORT method. Gene Set Variation Analysis (GSVA) was conducted to compare the biological pathways involved in the low-risk and high-risk groups. Furthermore, paraffin sections of HCC tissue microarrays containing 77 patients from Fudan University Shanghai Cancer Center were used for IHC staining. The clinical characteristics of the 77 HCC patients were collected and summarized for survival analysis validation *via* the Kaplan–Meier (KM) method.

**Results:** Three-gene signature with close immune correlation (Risk score = EPO * 0.02838 + BIRC5 * 0.02477 + SPP1 * 0.0002044) was constructed eventually and proven to be an effective prognostic factor for HCC patients. The patients were divided into a high-risk and a low-risk group according to the optimal cutoff, and the survival analysis revealed that HCC samples with high-risk immuno-score had significantly poorer outcomes than the low-risk group (*p* < 0.0001). The results of CIBERSORT suggested that the immune cell activation was relatively higher in the low-risk group with better prognosis. Besides, GSVA analysis showed multiple signaling differences between the high- and low-risk group, indicating that the three-gene prognostic model can affect the prognosis of patients by affecting immune-related mechanisms. Tissue microarray (TMA) results further confirmed that the expression of three genes in HCC tissues was closely related to the prognosis of patients, respectively.

**Conclusion:** In this study, we constructed and validated a robust three-gene signature with close immune correlation in HCC, which presented a reliable performance in the prediction of HCC patients’ survival.

## Introduction

Primary liver cancer is one of the most common malignancy worldwide, ranking the top three in leading cause of cancer death in 2020 for both sexes (Sung et al., 2021). Hepatocellular carcinoma (HCC), the major histopathology type of primary liver cancer (accounting for 75%–85% of all cases), has poor long-term outcomes due to its occult incidence and difficulty of early diagnosis (Marrero et al., 2018; Llovet et al., 2021a). Though present common therapeutic strategies such as surgical resection, transplantation, chemotherapy, targeted therapy, and local treatment, among others, have certain survival benefits, lack of effective prognostic biomarkers and untimely diagnosis still result in unfavorable prognosis (European Association for the Study of the Liver, 2018; Llovet et al., 2018; Yoh et al., 2021). Moreover, HCC patients with the same TNM (Tumor Node Metastasis) stage could experience different prognosis, indicating that better prognostic factors are urgently needed for treatment guiding and prognosis predicting. With the deepening understanding of immune microenvironment and intratumor heterogeneity in HCC, the treatment of HCC has entered a new era of immunotherapy (Kurebayashi et al., 2018). Mounting evidence revealed that immune checkpoint inhibitors (ICIs) could increase the response rate of advanced HCC patients and enhance the efficiency of radiotherapy and locoregional therapies (Llovet et al., 2021b; Cheu and Wong, 2021). However, only sectional HCC patients react to the current immunotherapy, experiencing prolonged survival. Therefore, studies focused on investigating potential immune targets and robust prognostic markers are urgently needed.

The tumor microenvironment (TME) is a complex ecology harboring diverse cell types, secreted cytokines, and extracellular matrix (ECM), which plays an important role in carcinogenesis and therapeutic resistance of malignancies (Bejarano et al., 2021). As a central immunological organ, liver exposes a plethora of immune cells from the adaptive immune system and the innate immune system (Leone et al., 2021). To maintain homeostasis, the liver forms a tolerogenic microenvironment to prevent constant inflammation and tissue damage, making HCC a type of heterogeneous inflammation-driven malignancy with complex immune tolerance microenvironment (Ruf et al., 2021). Because of the unique immune landscape of liver, immunotherapy for HCC is particularly challenging. Thus, identification of novel markers targeting TME for immunotherapies is particularly necessary. Nevertheless, research focusing on immune-related prognostic gene models in HCC have been inadequate and lack accurate validation. Presently, with the continuous development of bioinformatics, a variety of new algorithms emerge, providing a brand-new platform and approach for oncology precision medicine research. Integrative analysis based on these abundant processed high-throughput data may provide new discovery and insight that facilitating HCC immunotherapies.

This study aimed to construct a robust gene signature with close immune correlation for prognosis prediction of HCC. Overlapping immune-related genes (IRGs) from TCGA-LIHC and Immunology Database and Analysis Portal (ImmPort) website were analyzed to identify prognosis-related candidate genes. LASSO-penalty regression analysis was conducted to acquire three key genes to build the risk signature. CIBERSORT and GSVA were utilized to explore the potential biological functions of the identified IRG signature. Moreover, an HCC tissue microarray (TMA) containing 77 patients were used for IHC staining to verify the predictive capability of the signature. The results proved the significant role of the identified three-gene signature in HCC diagnosis and prognosis prediction, providing new insights for immunotherapies development in HCC.

## Materials and Methods

### Data Source and Preparation

In this study, FPKM-normalized transcriptome profiling data of The Cancer Genome Atlas Liver Hepatocellular Carcinoma (TCGA-LIHC) from the Genomic Data Commons (GDC) portal (https://portal.gdc.cancer.gov) were downloaded and used as the training set for model construction. For the standardization of data from TCGA-LIHC, a log transformation of each gene was performed by the formula of log[(expression level + 1), 2]. To circumvent the issue of undefined log(0), a “pseudocount” + 1 was added (Booeshaghi and Pachter, 2021).

### Immune-Related Genes Extraction

A total of 1,811 IRGs were downloaded from the Immunology Database and Analysis Portal (ImmPort) website (https://www.immport.org). After intersecting with TCGA data, 1,474 overlapping IRGs with a detection rate of more than 10% were selected for the following analysis.

### Construction and Verification of the Prognostic Model

After standardizing the data of TCGA-LIHC log[(expression level+1), 2], the Least Absolute Shrinkage and Selection Operator (LASSO) regression analysis was conducted according to the corresponding expression value of these samples and the clinical condition of prognosis. LASSO is an appropriate high-dimensional regression classifier that could be extended to Cox proportional hazard regression model for prognostic analysis. We used LASSO Cox regression model to predict the most relevant marker for prognosis based on 364 samples by using the “glmnet” R package (version 3.3.1) (Friedman et al., 2010; Simon et al., 2011). Identified genes were entered into a Cox proportional hazard regression model, followed by a leave-one-out/10-fold cross-validation to avoid model overfitting. In LASSO regression, the optimal value of the penalty parameter (*λ*) was finalized using the lambda.min, corresponding to the minimum of the partial likelihood deviance.

The risk score evaluation formula was constructed according to the three markers to make a high-risk and low-risk assessment of these samples. Thus, the risk score of each sample in the training set was calculated using the following formula:

$$\mathrm{Riskscore}=\overset{n}{\underset{i=1}{\sum }}\left(\omega_{i}*\chi_{i}\right)$$

Where *ω*_*i*_ is the coefficient of each gene obtained from the LASSO Cox regression analysis, and χ_*i*_ is the expression value of each gene.

We selected the best cutoff value in the training set using the X-tile plots (Camp et al., 2004) and applied this value to classify the HCC patients. The patients whose risk score was higher than the best cutoff risk score were classified into the high-risk group, and those whose risk score was lower than the best cutoff were classified into the low-risk group. The Kaplan–Meier (KM) survival analysis with the log-rank method was applied to evaluate the association of the risk score with prognosis. Survival analysis was conducted using the “survival” R package to compare the predicted and observed overall survival (OS) between the two subgroups. Time-dependent receiver operator characteristic (ROC) curve analysis was applied to verify the sensitivity and predictive ability of the risk score in HCC using the R packages “survivalROC” (Lorent et al., 2014).

### Immune Correlation Analysis

The CIBERSORT method was applied to estimate the immune infiltration of the 364 HCC samples using the R package “cibersort” (Chen et al., 2018). Based on the transcriptome expression data, the relative expression level of the 22 tumor infiltration leukocytes (TILs) was quantified in high-risk and low-risk subgroups to investigate the impact of risk score on the tumor immune environment. Normalized enrichment scores were calculated for each immune cell type with the deconvolution approach application, and only samples with *p*-value < 0.05 were included for analysis. Pearson correlation coefficient was used to compare the similarity of signature genes in different mixtures. Root mean square error (RMSE) was obtained to measure the deviation between the observed value and the real value. A two-sided Wilcoxon test was used to determine the differences in immune cell subtypes between the high-risk and low-risk group.

### Gene Set Variation Analysis

Gene Set Variation Analysis (GSVA), a gene set enrichment method that could estimate pathway activity variation over a sample population in an unsupervised manner, shows a stronger ability to deal with molecular mapping experiments (Hänzelmann et al., 2013). Using the R package “GSVA”, the enrichment score of different biological pathways in each sample was calculated. Through GSVA analysis of different genes in the low-risk group and high-risk group, the variation of related pathways in different states was obtained. Differentially enriched pathways between the two groups were further identified with the threshold of false discovery rate (FDR) < 0.01 and *p*-value < 0.01.

### Human HCC Sample Collection

Formalin-fixed paraffin-embedded (FFPE) sections of HCC tissue microarrays containing 77 HCC patients from Fudan University Shanghai Cancer Center were used for IHC staining. All patients or their families obtained informed consent with details of this study provided. The current study was approved by the Ethics Committee of the Fudan University Shanghai Cancer Center. The clinicopathologic data and prognostic status of each patient were collected for further analysis.

### Immunohistochemical Staining of the Tissue Microarray

Sections were stained with primary antibodies against EPO (Rabbit Polyclonal, Catalog number: 17908-1-AP, Proteintech Group, Chicago, IL, United States), BIRC5 (Rabbit mAb #2808, 71G4B7, Cell Signaling Technology), and SPP1 (Rabbit Polyclonal, Catalog number: 22952-1-AP, Proteintech Group, Chicago, IL, United States). IHC staining score can be divided into two parts: staining intensity and percentage of positive tumor cells. Staining intensity was classified as 0 (no staining), 1 (weak staining), 2 (moderate staining), and 3 (strong staining). Percentage of positive tumor cells was classified as 1+ (≤25%), 2+ (26%–50%), 3+ (51%–75%), and 4+ (>75%). The final expression scores were calculated by multiplying the two variables together. With a maximum score of 12, all samples were further divided into a low expression group and a high expression group according to the moderate score of 6 (≥6: high expression; <6: low expression). Three pathologists independently evaluated the staining results.

### Statistical Analysis

All statistical analysis and graph plotting were performed using R software (Version 3.3.1; R Foundation for Statistical Computing, Vienna, Austria), SPSS 25.0 (IBM, New York, United States), and GraphPad Prism 8.0 (GraphPad Software, San Diego, CA, United States). Log-rank test was used to show survival differences in the Kaplan–Meier curves to compare the OS between the two groups. The area under the ROC curve (AUC) was calculated to determine the sensitivity and prognostic performance of the identified survival predicting model. Mann–Whitney test was used to compare immune cell infiltration between subgroups. The boxplots of two groups were analyzed using Wilcoxon test. *p*-value < 0.05 was considered statistically significant.

## Results

### Study Design and Subjects

**FIGURE 1:**
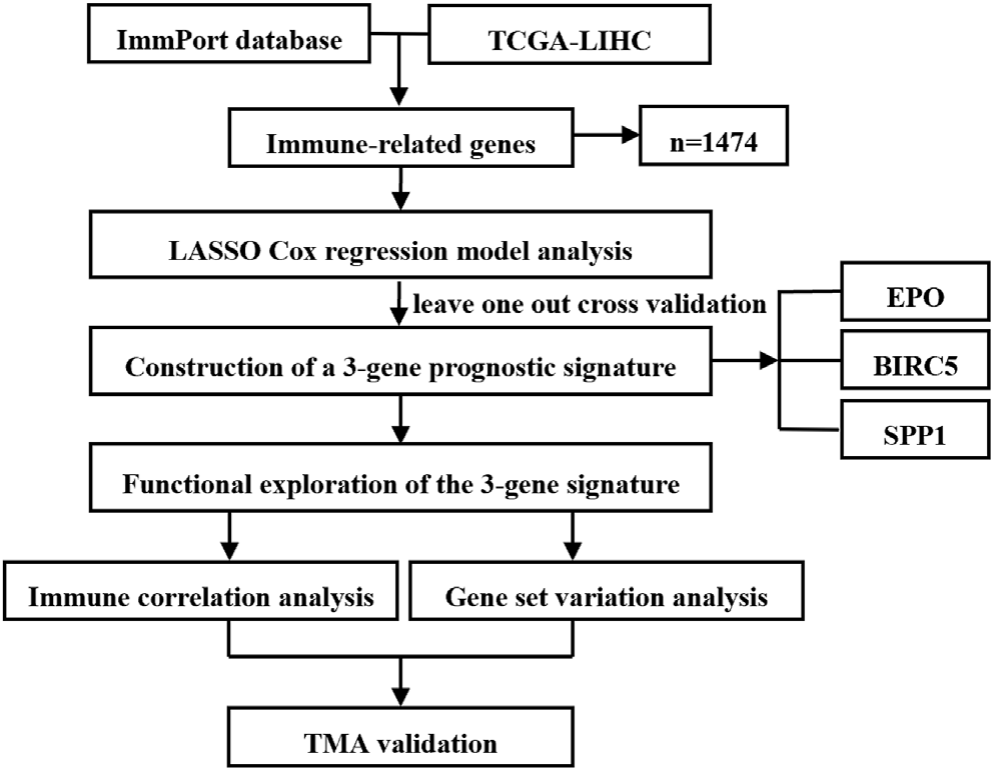
Flow chart of study design.

The flow chart of study design is shown in Figure 1. A total of 364 HCC samples with follow-up information in the TCGA-LIHC dataset were analyzed for the training of the prognostic signature. The correlation between the identified gene signature and immune status was further explored in the TCGA dataset. Immune correlation analysis and GSVA were conducted for functional exploration of the three-gene prognostic model. The clinical characteristics of the 77 HCC patients from Fudan University Shanghai Cancer Center were collected and summarized for survival analysis validation combining TMA staining results.

### Identification of Immune-Related Genes

We intersected the IRGs from the ImmPort database with the data of TCGA-LIHC to further screen the IRGs involved in the occurrence and development of HCC. A total of 1,474 IRGs with a detection rate of more than 10% were finally obtained for further analysis, and more detailed information of gene expression can be found in Supplementary Material S1 **.**

### Development of Prognostic Model Based on Survival Related IRGs

**FIGURE 2:**
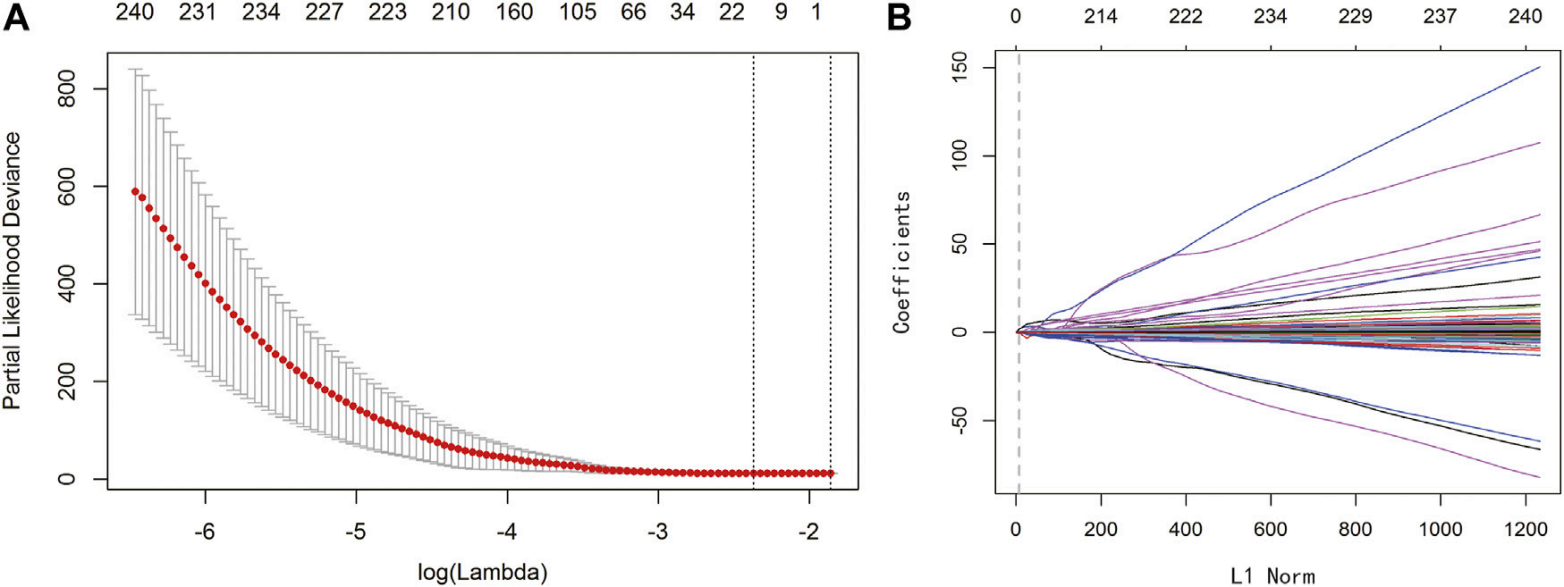
LASSO regression analysis identified three significant genes. **(A)** LASSO deviance profiles. The *y*-axis represents partial likelihood deviation. The *x*-axis represents the ideal gene feature amount on various lambda values. The two longitudinal dotted line indicates λmin **(left)** and λ1‐SE **(right)**, respectively. **(B)** LASSO coefficient profiles. The *y*-axis represents coefficients, and the *x*-axis represents L1 Norm. The vertical dotted line indicates that variables intersecting the corresponding penalty value are finally included in the model, and the corresponding ordinates of the variables are the weighting coefficients.

To establish a clinically applicable risk assessment model, LASSO regression analysis was conducted to evaluate the identified 1,474 IRGs combined with the 364 samples with expression profiles in TCGA-LIHC to minimize overfitting, and the R package “glmnet” was used to find the best gene signature. Finally, three key genes were identified as prognostic markers for HCC patients *via* leave-one-out cross-validation method (EPO: Erythropoietin; BIRC5: Baculoviral IAP repeat-containing protein 5; SPP1: Secreted phosphoprotein 1). The optimal lambda value in LASSO model is shown in Figure 2A, and the regression coefficient of the three key genes in HCC is presented in Figure 2B. The biological functions and marker risk coefficients of key genes are listed in Table 1. The predicted immunoscore can be calculated with the following formula: 0.02838 * EPO + 0.02477 * BIRC5 + 0.0002044 * SPP1.

**TABLE 1:** Biological functions and marker coefficient of key genes.

Gene symbol	Full name	Function	Coef	*p*-value
EPO	Erythropoietin	Regulating erythrocyte proliferation and differentiation	0.02838	0.000708
BIRC5	Baculoviral IAP repeat-containing protein 5	Promoting cell proliferation and preventing apoptosis	0.02477	0.00048
SPP1	Secreted phosphoprotein 1	Essential cytokine in the pathway that leads to type I immunity	0.0002044	0.001337

### Validation of the Predictive Capability of the Three-Gene Risk Signature

**FIGURE 3:**
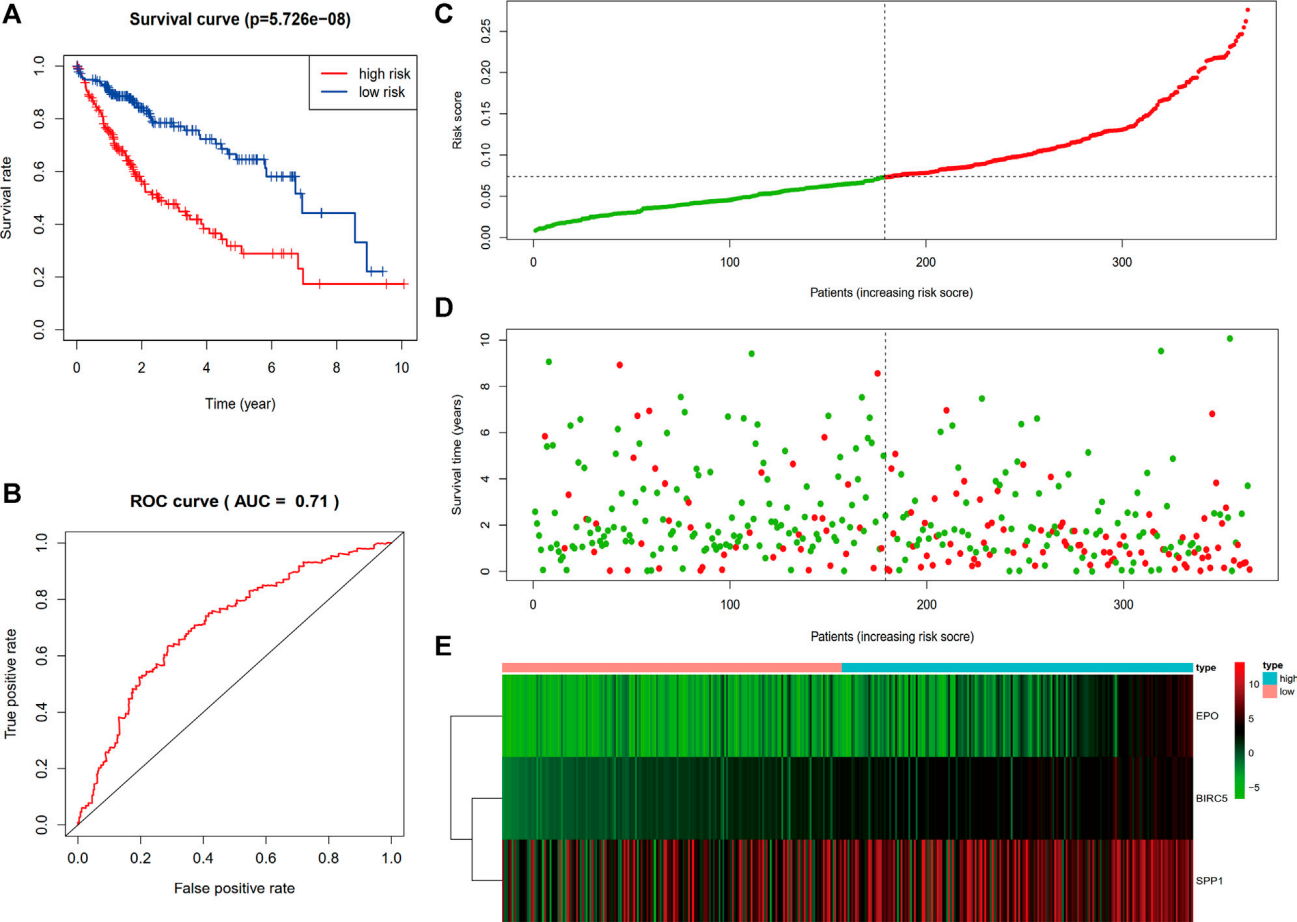
Evaluation and validation of the prediction value of the three-gene risk signature. **(A)** Kaplan–Meier survival curve of the risk score. **(B)** ROC curve comparing the prognostic values of risk score. **(C)** Distribution plot of risk score. **(D)** Survival status of each patient. **(E)** Heatmap of mRNA expression levels of the three genes.

The individualized immune risk score of each patient was calculated according to the corresponding coefficients, and the optimal cutoff of 0.0732 was determined by the X-tile software. Subsequently, the enrolled 364 TCGA-LIHC samples with survival profiles were stratified into high-risk (*n* = 185) and low-risk (*n* = 179) groups (Supplementary Material S2). To further verify the predictive power of the immune-correlated three-gene signature, the KM curve analysis and time-dependent ROC curves were conducted to compare the difference between the low-risk and high-risk group. Figure 3A showed that the high-risk group performed a poorer OS compared to the low-risk group in KM curve analysis calculated *via* Log Rank (Mantel–Cox) (*p* < 0.0001). Moreover, the area under the curve of ROC (AUC) for the OS predictions of risk score was 0.71 (Figure 3B), indicating the strong predictive power of the identified three-gene signature. Then, the distribution plot of risk score was depicted and showed more deaths in the high-risk group (Figure 3C). Survival status of each patient in Figure 3D showed better prognosis in the low-risk group (Figure 3D). The heatmap of mRNA expression levels of the three genes is shown in Figure 3E. The above results suggested that the three-gene immune-related signature we built was verified to possess a good predictive ability on the survival of HCC patients.

### Relationship Between the Immune-Related Gene Signature and Clinical Characteristics

**FIGURE 4:**
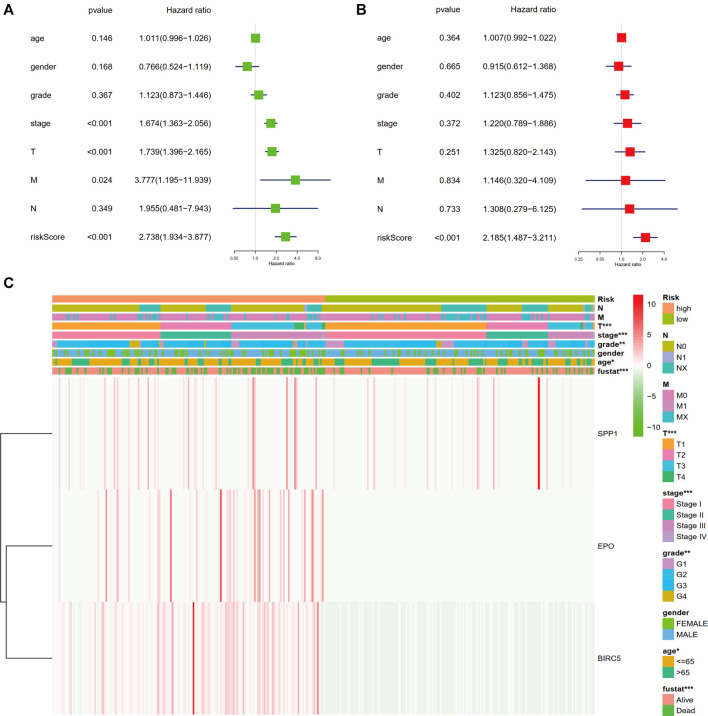
Relationship of the three-gene signature and clinical variables. **(A)** Univariate Cox proportional hazard regression analysis between the risk factors and overall survival of HCC patients. **(B)** Multivariate Cox proportional hazard regression analysis between the risk factors and overall survival of HCC patients. **(C)** Heatmap of the correlation analysis between gene signature and clinical pathological parameters of the HCC patients in the training set (*p < 0.05, **p < 0.01, ***p < 0.001).

We assessed the correlation between the three-gene signature and the clinical pathological parameters of the HCC patients in the training set. As Figure 4C showed, advanced T stage, clinical stage, histological grade, and age were found to be significantly related to higher risk score. Nevertheless, there is no difference between gender, N stage, and M stage. Moreover, univariate and multivariate Cox proportional hazard regression analysis between the risk factors and overall survival of HCC patients were performed to testify the role of the identified signature being an independent prognostic factor. In univariate analysis, we could see that the risk score, clinical stage, T stage, and M stage were correlated with OS of HCC patients (Figure 4A). The multivariate analysis results showed that only risk score could predict HCC patients’ disease outcomes independently (Figure 4B). The results indicated that our three-gene signature could be an independent prognostic factor in both univariable and multivariable Cox regression analyses.

### Immune-Relevance Analysis of the Prognostic Model

**FIGURE 5:**
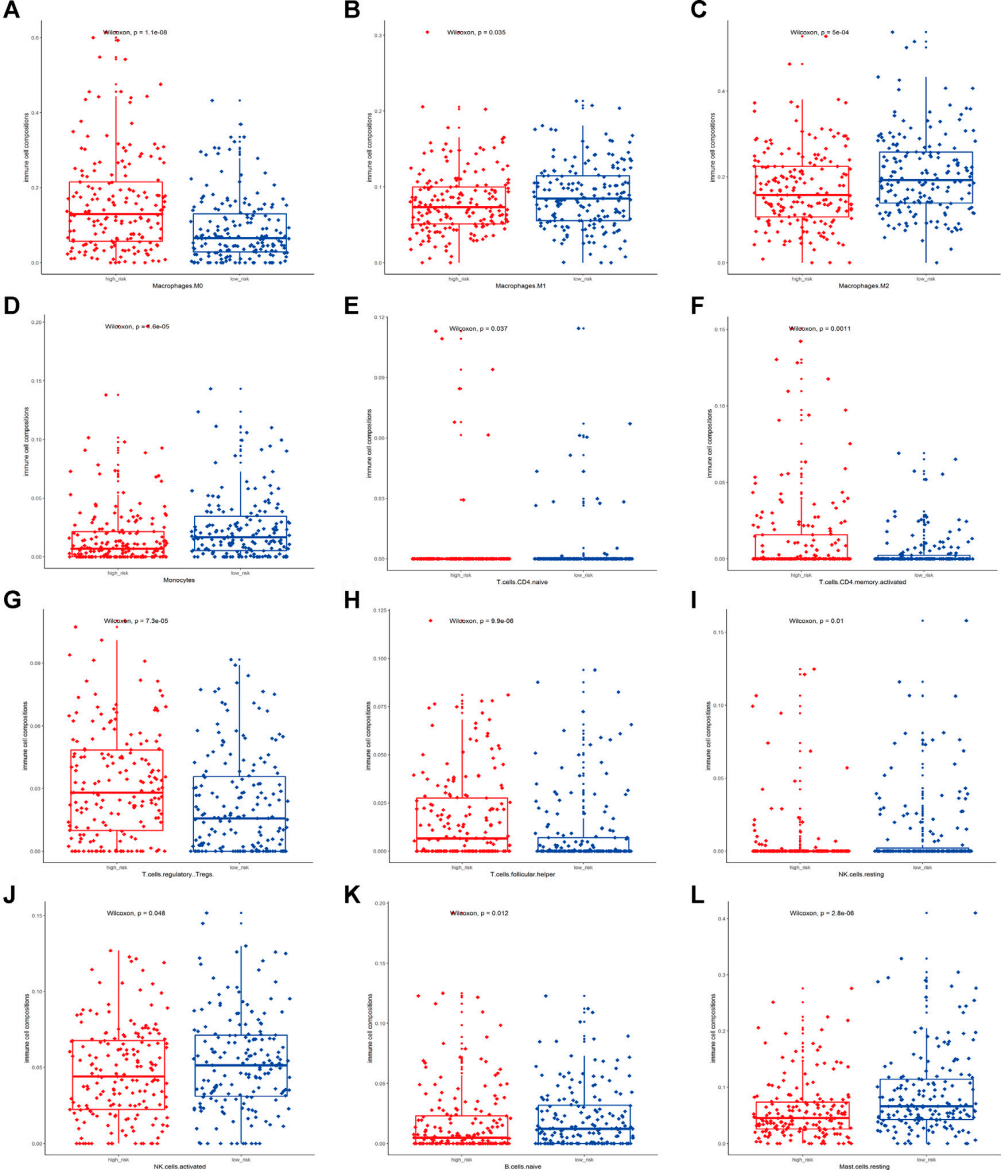
Association between the risk score of the signature and immune cell infiltration. **(A–L)** presented relative expression of M0 macrophages, M1 macrophages, M2 macrophages, Monocytes, naïve CD4^+^ T cells, activated CD4^+^ memory T cells, regulatory T cells (Tregs), follicular Helper T cells (Tfh), resting NK cells, activated NK cells, naïve B cells, and resting Mast cells (*p* < 0.05) between the low-risk and high-risk group, respectively.

To elucidate the association between the TME and the constructed three-gene signature, the CIBERSORT method was applied to quantify 22 types of tumor-infiltrating immune cells in 364 TCGA-LIHC samples (Supplementary Material S3). Figure 5 presents 12 cell types with significant expression difference between the high-risk and low-risk group (*p* < 0.05). The estimated proportions of M0 macrophages, activated CD4^+^ memory T cells, regulatory T cells (Tregs), and follicular Helper T cells (Tfh) were mainly enriched in the high-risk group. On the contrary, the low-risk group performed larger abundance of M1 macrophages, M2 macrophages, Monocytes, naïve CD4^+^ T cells, resting NK cells, activated NK cells, naïve B cells, and resting Mast cells (Figure 5). Meanwhile, 10 types of immune cells with no significant difference in expression (*p* > 0.05) are shown in Supplementary Figure S1. These results revealed the mutual correlation between the identified three-gene signature, immune relevance, and disease prognosis in HCC patients. Activation of immune cells was positively related to better disease outcomes, which deserve further exploration.

### GSVA of Differentially Activated Pathways

**FIGURE 6:**
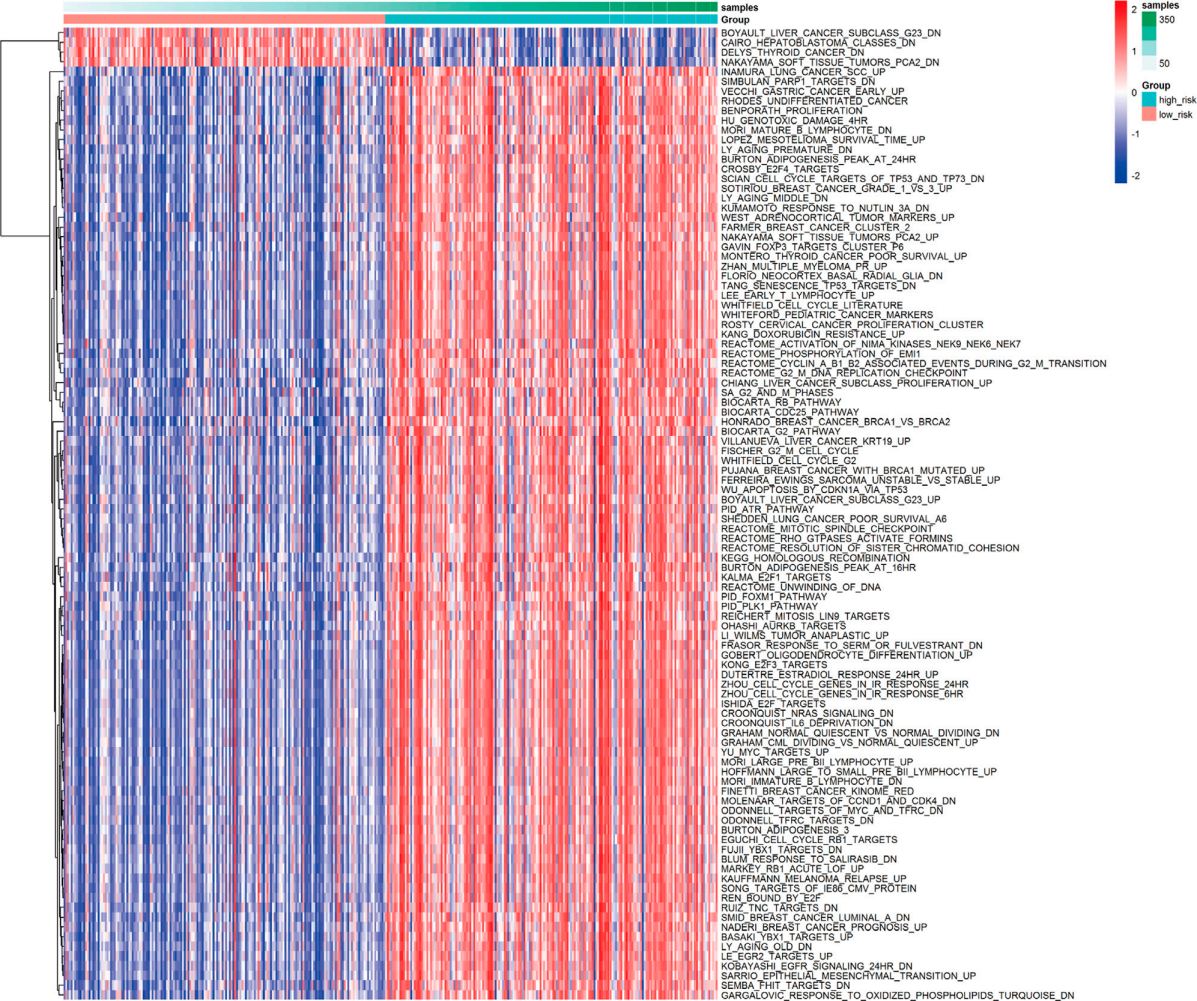
Different pathways between the high-risk and low-risk groups.

**FIGURE 7:**
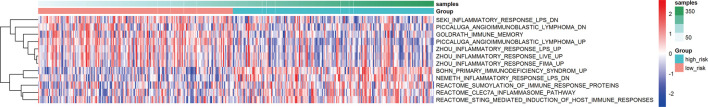
Variation of immune and inflammation-related pathways.

Based on the calculated enrichment score of each sample, we identified enriched-pathway variation between the low-risk and high-risk group *via* the GSVA method (FDR< 0.01, *p*-value < 0.01). The analysis details can be found in Supplementary Material S4, and the worksheet “path to genes” showed the corresponding genes in each pathway affected by the risk grouping, indicating the downstream of the three-gene model. Figure 6 shows the biological pathway gathering difference of 364 samples in the low-risk group and high-risk group. The color in the picture changes from blue to red, indicating an increase in the value of enriched score. From the low-risk to the high-risk group, the enrichment score was obviously increased in INMURA_LUNG_CANCER_SCC_UP, SIMBULAN_PARP1_TARGETS_DN, VECCHI_GASTRIC_CANCER_EARLY_UP, RHODES_UNDIFFERENTIATED_CANCER, BENPORATH_PROLIFERATION, HU_GENOTOXIC_DAMAGE_4HR, and others. The results of enrichment implied multiple pathway variation that deserves further exploration. The enrichment of MONTERO_THYROID_CANCER_POOR_SURVIVAL_UP in the high-risk group suggested that prognostic grouping of our gene signature was consistent with the prognosis of other tumors. CROONQUIST_IL6_DEPRIVATION_DN increased gradually from the low-risk to the high-risk group, indicating that there was a high expression of genes inhibited by the IL6-related pathway in the high-risk group, resulting in a lower survival rate. Then, we screened the immune and inflammation-related pathways, and found that with the increase of risk value, the enrichment of immune-related pathways gradually decreased (BOHN_PRIMARY_IMMUNODEFICIENCY_SYNDROM), while the inflammation-related pathways gradually upregulated (NEMETH_INFLAMMATORY_RESPONSE_LPS_DN), indicating that the grouping model composed of these three genes could affect immune-related mechanisms and closely related to prognosis (Figure 7).

### TMA Reaction Intensity Evaluation and Biomarkers Prognostic Value Validation

**FIGURE 8:**
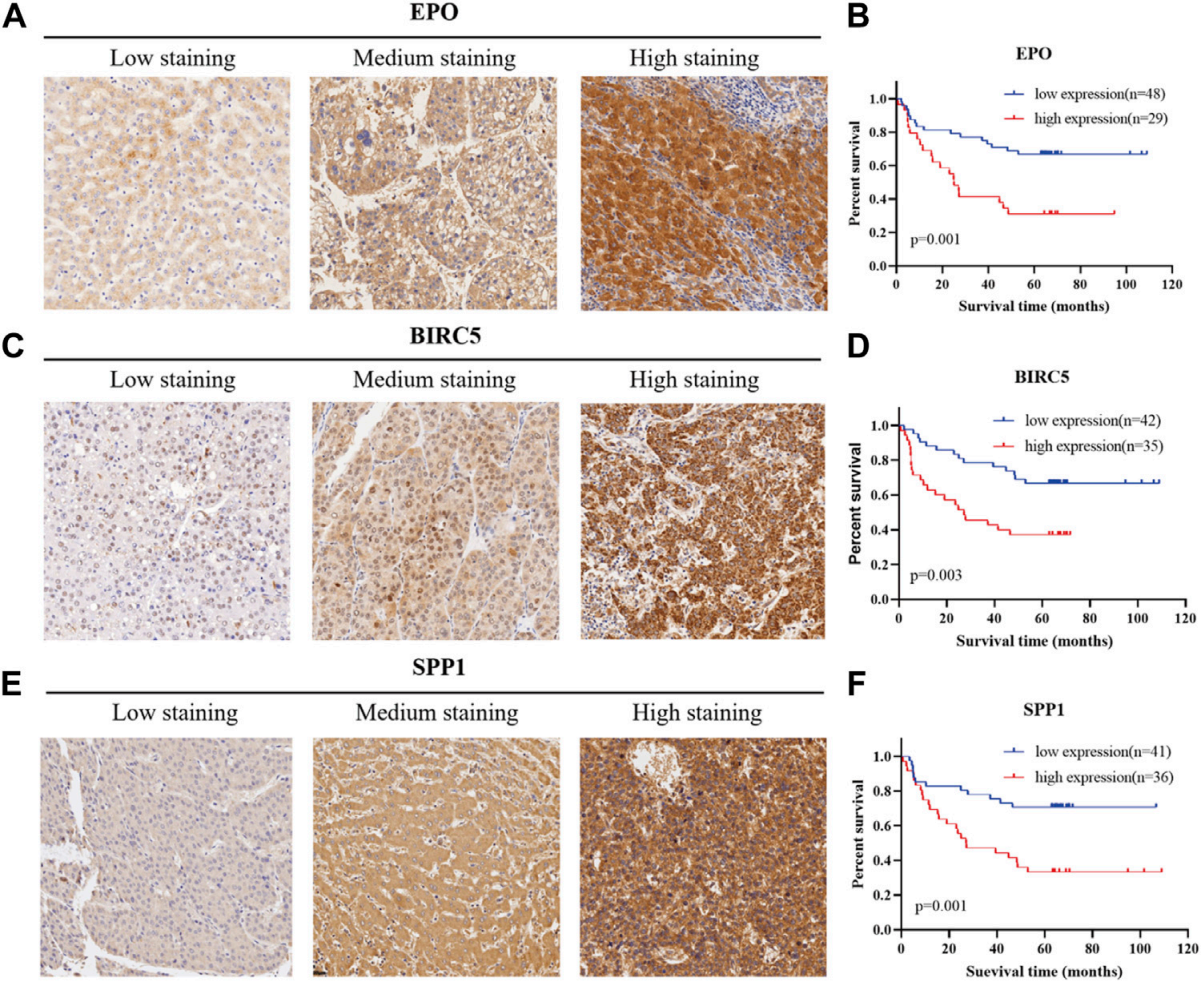
Verification of the prognostic efficiency of the three markers in HCC using a tissue microarray. **(A)**, **(C)**, **(E)** Representative immunohistochemistry images in HCC tissues of EPO, BIRC5, and SPP1 (200X).**(B)**, **(D)**, **(F)** Kaplan–Meier curves for overall survival of 77 HCC patients with different protein expression levels of EPO, BIRC5, and SPP1.

In order to verify the actual dependability and efficiency of the three-gene signature constructed from the bioinformatics analysis, we determined the protein level of the three genes in a TMA containing 77 HCC patients from Fudan University Shanghai Cancer Center. All 77 HCC patients had undergone complete surgical resection of the liver tumor with detailed pathological diagnosis reports between October 2011 and June 2015. Follow-up information collection for all patients was completed from the date of surgery until March 2021. Based on the staining score calculation formula mentioned above, we separated 77 patients into a high-expression and a low-expression group according to the histological score. Representative immunohistochemistry images with different staining intensity are shown in Figure 8A,C,E. Kaplan–Meier curves were used to explore whether the marker expression related to survival status and prognosis. As presented in Figure 8B, 48 patients were included in the EPO low-expression group and 29 patients in the high-expression group. The statistical analysis indicated that high EPO expression could predict worse disease outcomes (*p* = 0.001). Similarly, high expression of BIRC5 and SPP1 proved to have a close correlation with poor prognosis (*p* = 0.003, *p* = 0.01, Figures 8D,F). These results indicated the actual prognostic value of the key markers in the identified gene signature.

## Discussion

HCC, representing the most common prototype of primary liver cancer, is traditionally considered as a kind of inflammation-driven and chemotherapy-resistant entity. Despite the advances in therapeutic managements, advanced HCC patients still possess poor outcomes due to its delayed diagnosis and limited treatment choices (Marrero et al., 2018). Nowadays, immunotherapies have become novel pillars and avenues for cancer treatment (Binnewies et al., 2018). Emerging studies indicated that the cross talk between liver immune microenvironment and tumor cells played an important role in promoting hepatocarcinogenesis (Cao et al., 2020; Yasuoka et al., 2020). Clinically, recent literatures have proven the potential benefits of implementing immunotherapies in HCC (Kudo, 2019; Jiang et al., 2020a; Nishida and Kudo, 2020). Nevertheless, only a small portion of HCC patients could have durable responses to current immunotherapies. To solve the stalemate, it is crucial to analyze the different immune status of HCC patients to find efficient diagnostic biomarkers.

Therefore, the purpose of our study was to screen IRGs in HCC, and build an appropriate gene signature with valuable predicting capability of disease outcomes. After intersecting the data from TCGA-LIHC and ImmPort website, we selected 1,474 overlapping IRGs for LASSO regression analysis. Eventually, a three-gene signature (EPO, BIRC5, and SPP1) was constructed by random sampling of the leave-one-out cross-validation method. According to the established formula, we stratified HCC patients into the high-risk and low-risk groups. The survival analysis showed that the high-risk group had worse prognosis compared to the low-risk group.

Erythropoietin (EPO), a glycoprotein hormone stimulator of erythropoiesis, could regulate erythrocyte proliferation and differentiation (Lacombe and Mayeux, 1998). Erythropoietin/erythropoietin-receptor system has been proven to be involved in angiogenesis in HCC (Ribatti et al., 2007). Recent studies demonstrated that EPO was upregulated in human HCC tissues and promoted the proliferation of HCC through hypoxia-induced translocation of its specific receptor (Miao et al., 2017). Qiu et al. reported that EPO-positive patients showed poorer prognosis for OS than EPO-negative patients, which was consistent with what we observed (Yang et al., 2015). Moreover, EPO has been found to be associated with redox-immune and hypoxia in HCC, indicating that EPO is a potential therapeutic target for HCC (Jiang et al., 2020b; Tu et al., 2021). Baculoviral IAP repeat-containing protein 5 (BIRC5), also known as survivin, is an inhibitor of apoptosis (IAP) protein, which could promote cell proliferation and prevent apoptosis (Li et al., 1998). BIRC5 expression could reflect aggressive histological and clinical behavior of HCC, correlating with poorer OS (Kapiris et al., 2019). Present studies have shown that BIRC5 is closely related with autophagy, and several survival indexes containing BIRC5 were proved to have promising predictive value (Yang et al., 2020; Qin et al., 2021; Wang et al., 2021). Jiao et al. once reported that YAP promoted sorafenib resistance in HCC by upregulating BIRC5 (Sun et al., 2021). According to Chang et al., BIRC5 may play a vital role in the IGF-1 signaling pathway by mediating EMT in HCC (Liu et al., 2018). Wang et al. reported a novel prognostic index containing BIRC5 as a key factor in HCC, which reflected the infiltration of a variety of immune cells (Xiao et al., 2021). Secreted phosphoprotein 1 (SPP1), encoding osteopontin (OPN), acts as an essential cytokine in the pathway leading to type I immunity and cell-matrix interaction. In the liver, OPN was found to contribute to fibrogenesis (Urtasun et al., 2012). Beretta et al. identified that plasma OPN was significantly elevated in HCC patients, demonstrating that OPN could be a marker for early HCC (Shang et al., 2012). Hsu et al. reported that OPN overexpression is associated with intrahepatic metastasis and early recurrence of surgically resected HCC, indicating a poorer prognosis (Pan et al., 2003). Another study by Abdel-Hafiz et al. confirmed the close relation between HCC and OPN serum concentrations (Abdel-Hafiz et al., 2018), which was also consistent with our findings.

Since the immune cell infiltration of TME *in situ* was considered as a valuable index for disease outcomes prediction and immunotherapy response in malignancies, it is necessary to explore and illustrate the association between HCC immune cell infiltration and the identified gene signature to display the HCC TME status. In this study, we estimated the abundance of 22 different types of immune cells in HCC sample *via* the CIBERSORT algorithm. According to our results, the high-risk group tended to have less immune cell infiltration, indicating that activation of immune cells may positively correlate with better prognosis. The results showed that Tregs, Tfh, M0 macrophages, and activated CD4^+^ memory T cells were more enriched in the high-risk group. Tregs’ potent suppressing function promotes immune tolerance *via* various mechanisms. In HCC, a higher density of Tregs intratumorally or in peripheral blood compared with CD8^+^ T cells indicated a worse prognosis (Fu et al., 2007). Spengler et al. found that Tregs contributed to systemic immune dysfunction and facilitated tumor progression (Langhans et al., 2019). Additionally, Tfh was reported to be correlated with the progression of HCC patients (Yuan et al., 2021). Previous studies revealed that Tfh cells moved into periphery in cancer and inflammation by downregulating CXCR13 expression (Dieu-Nosjean et al., 2016). Guo et al. also found that tumor-induced inflammation could stimulate Tfh cells (Wang et al., 2020). As a kind of resting macrophage, M0 macrophage was considered to be related to immune functional inhibition. According to Chi et al., the infiltrating percentage of M0 macrophages in HCC tumor tissue was significantly higher than that of normal tissue, consistent with our findings (Yang et al., 2021a). Moreover, previous lines of evidence showed that activated CD4^+^ memory T cells played an important role in Type 1 immune responses (Krueger et al., 2021) and was correlated with inflammation (Fanelli et al., 2021). Xu et al. also reported activated CD4^+^ memory T-cell infiltration related to high tumor mutational burden (TMB) in HCC (Yin et al., 2020). As a result, our preliminary findings may provide an orientation to exploit the deeper correlation between immune infiltration an HCC.

Besides, we analyzed the pathway variation based on the identified enrichment score *via* GSVA. The results showed that the risk score calculated according to the signature was positively correlated with the enrichment of inflammation-related pathways. Meanwhile, immune-related pathways were significantly enriched in low-risk groups. Previous studies reported that involved in macrophage activation and DNA damage, reactive oxygen species and oxidative stress were tightly linked to inflammation and carcinogenesis (Leone et al., 2021). Shan et al. observed that the expression of Oxidative stress-responsive 1 (OXSR1) was abnormally elevated in HCC, and confirmed as an independent prognostic factor in HCC patients (Chen et al., 2020). Moreover, as the GSVA results indicated, PARP1 has been proven to be overexpressed in HCC patients and closely related to poor prognosis (Yang et al., 2021b). Meanwhile, the GSVA results showed that genes related to interleukin 6 (IL-6) deprivation pathway were highly enriched in the high-risk group. In fact, many studies have shown that these immune cells are closely associated with IL-6. Hackl et al. reported that IL-6 produced by DCs could mediate the inhibitory effect of the Toll-like receptor 7 (TLR7) ligand on Treg cell generation (Hackl et al., 2011). Jones et al. reported that IL-6 produced by the murine activated DCs was essential for CD4^+^ T-cell expansion, enhancing the response of aged naive CD4 T cells (Jones et al., 2010). Ma et al. revealed that the ability of IL-6 to promote humoral immunity was correlated to its effects on Tfh (Ma et al., 2012).The exact roles of these genes and their correlated pathways in HCC immunity deserve further investigation in the future.

In summary, we identified and validated a three-gene signature with close immune correlation for the survival prediction of HCC. This work complemented the deficiency of the existing liver cancer staging system in guiding the prognosis, providing a new perspective for the future study of HCC immunotherapy and TME. Nevertheless, there were still certain limitations in this study that should be noted. Firstly, this study was retrospective, and further verification in clinical trials should be conducted before clinical application. Secondly, due to the missing information during the follow-up process, we only analyzed overall survival instead of progression-free survival, which limited the maximum use of statistics. The sample size can be further expanded as well. Meanwhile, in order to investigate the exact molecular mechanisms and biological function of the three-gene signature, further experimental validation should be additionally performed.

## Conclusion

In this study, a robust three-gene signature with close immune correlation in HCC was constructed and validated. This identified novel risk model exhibited reliable performance in the prediction of HCC patients’ survival, which may contribute to the immunotherapy research for HCC, deserving further exploration for better prognostic stratification and clinical practice.

## Data Availability

The original contributions presented in the study are included in the article/Supplementary Material. Further inquiries can be directed to the corresponding author.
