# Supplementary figures and images for: Identification and Validation of a Tumor Microenvironment-Related Gene Signature in Hepatocellular Carcinoma Prognosis
Source: Front Genet. 2021 Nov 26;12:717319. doi: 10.3389/fgene.2021.717319 (PMC8662347; doi:10.3389/fgene.2021.717319)

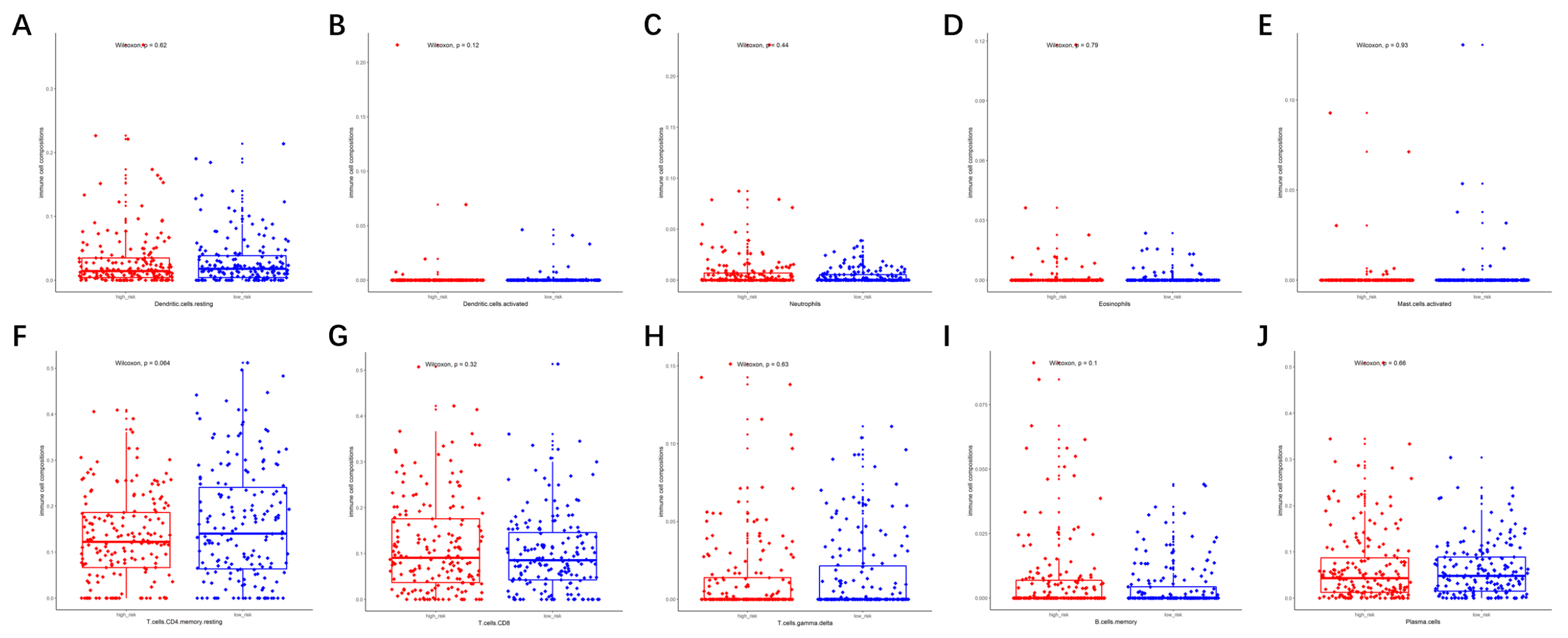

Supplement: Supplementary file 2 [file Image1.TIFF]
